# Oxytocin Protects Against Isoproterenol-Induced Cardiac Hypertrophy by Inhibiting PI3K/AKT Pathway via a lncRNA GAS5/miR-375-3p/KLF4-Dependent Mechanism

**DOI:** 10.3389/fphar.2021.766024

**Published:** 2021-12-03

**Authors:** Yuqiao Yang, Zhuoran Wang, Mengran Yao, Wei Xiong, Jun Wang, Yu Fang, Wei Yang, Haixia Jiang, Ning Song, Lan Liu, Jinqiao Qian

**Affiliations:** ^1^ Department of Anesthesiology, First Affiliated Hospital of Kunming Medical University, Kunming, China; ^2^ Department of Pathology, Kunming Medical University, Kunming, China

**Keywords:** oxytocin, cardiac hypertrophy, lncRNA GAS5, miR-375-3p, KLF4, PI3K/AKT

## Abstract

Cardiac hypertrophy is caused by cardiac volume or pressure overload conditions and ultimately leads to contractile dysfunction and heart failure. Oxytocin (OT), an endocrine nonapeptide, has been identified as a cardiovascular homeostatic hormone with anti-hypertrophic effects. However, the underlying mechanism remains elusive. In this study, we aimed to investigate the role and mechanism of OT in cardiac hypertrophy. The rats with cardiac hypertrophy induced by isoproterenol (ISO) were treated with or without oxytocin. Cardiac functional parameters were analyzed by echocardiography. The changes in cell surface area were observed using wheat germ agglutinin (WGA) or immunofluorescence staining. The expressions of cardiac hypertrophy markers (B-Natriuretic Peptide, BNP and β-myosin heavy chain, β-MHC), long non-coding RNA Growth (LcRNA) Arrest-Specific transcript 5 (lncRNA GAS5), miR-375-3p, and Kruppel-like factor 4 (*Klf4*) were detected by qRT-PCR. KLF4 protein and PI3K/AKT pathway related proteins were detected by Western blot. The interactions among lncRNA GAS5, miR-375-3p, and *Klf4* were verified by dual-luciferase reporter assays. The findings showed that OT significantly attenuated cardiac hypertrophy, increased expressions of lncRNA GAS5 and KLF4, and decreased miR-375-3p expression. *In vitro* studies demonstrated that either knock-down of lncRNA GAS5 or *Klf4*, or over-expression of miR-375-3p blunted the anti-hypertrophic effects of OT. Moreover, down-regulation of lncRNA GAS5 promoted the expression of miR-375-3p and inhibited KLF4 expression. Similarly, over-expression of miR-375-3p decreased the expression of KLF4. Dual-luciferase reporter assays validated that lncRNA GAS5 could sponge miR-375-3p and *Klf4* was a direct target gene of miR-375-3p. In addition, OT could inactivate PI3K/AKT pathway. The functional rescue experiments further identified OT regulated PI3K/AKT pathway through lncRNA GAS5/miR-375-3p/KLF4 axis. In summary, our study demonstrates that OT ameliorates cardiac hypertrophy by inhibiting PI3K/AKT pathway via lncRNA GAS5/miR-375-3p/KLF4 axis.

## Introduction

The prevalence of heart failure (HF) is increasing at an alarming rate worldwide (Benjamin et al., 2019). Pathological cardiac hypertrophy is an independent predictor of HF, commonly induced by hypertension, myocardial injury, valvular heart diseases or excessive neurohumoral stimulation. It is characterized by myocardial fibrosis, apoptosis and necrosis, leading to cardiac dysfunction and consequently to heart failure (Azevedo et al., 2016). Alleviating pathological cardiac hypertrophy is of great importance to postpone the progression of heart failure. Although the current pharmaceutical therapies, such as angiotensin-converting enzyme inhibitors, angiotensin II (AngII) type 1 receptor antagonists, and β-adrenergic receptor blockers, exhibit some anti-hypertrophic effects (Mcnamara, 2008), the therapeutic effects are not satisfied in clinic practice.

Oxytocin (OT) is a pivotal cardiovascular homeostatic hormone with definite cardiovascular regulation and protection effects. Knowledge of the oxytocin and oxytocin receptor (OXTR) system present in heart tissue suggests an autocrine and paracrine roles of the hormone. Dozens of researches have delineated the cardioprotective effects of this endogenous hormone, including alleviation of ischemia-reperfusion (I/R) injury (Alizadeh et al., 2010; Gonzalez-Reyes et al., 2015; Polshekan et al., 2019), cardiomyocyte hypertrophy (Menaouar et al., 2014), myocardial infarction (Kobayashi et al., 2009), and diabetic cardiomyopathy (Plante et al., 2015), as well as mitigation of development of atherosclerosis (Wang et al., 2019). The cardioprotective properties of oxytocin make this endogenous hormone of special potential to be a “natural medicine” against cardiovascular diseases. However, due to lack of deep knowledge of its molecular mechanism, therapeutic potential of OT in treating cardiovascular disease is largely unexplored.

Enormous studies have corroborated that non-coding RNAs (ncRNAs) are quite indispensable epigenetic regulators which are closely related to the occurrence and development of cardiac hypertrophy (Jusic and Devaux, 2020; Zhu et al., 2021). MicroRNAs (miRNA) and long non-coding RNAs (lncRNAs) are well-known ncRNAs and implicated in the complex pathophysiological process of cardiac hypertrophy. In the nervous system, Almansoub et al. (2020) reported that miR-26a is epigenetically tuned by oxytocin to mediate the neuroprotective effects. Whether OT administration regulates ncRNAs in heart is still unknown. Although the beneficial effects of OT in the prevention of pathological cardiac hypertrophy have been well documented (Menaouar et al., 2014; Plante et al., 2015; Garrott et al., 2017), the mechanisms responsible for these actions are unclear, especially the roles of non-coding RNAs (ncRNAs) in the effect.

LncRNA Growth Arrest-Specific transcript 5 (lncRNA GAS5) is identified as a novel regulator of hypertension-related vascular remodeling (Wang et al., 2016) and also closely associated with cardiac fibrosis and cardiomyocyte apoptosis, as evidenced by over-expression of lncRNA GAS5 significantly attenuates cardiac fibrosis (Liu et al., 2019) and cardiomyocyte apoptosis (Hao et al., 2018) induced by isoproterenol (ISO).

In our preliminary experiments, we detected that OT ameliorated cardiac hypertrophy induced by ISO along with lncRNA GAS5 remarkably upregulated, as well as miR-375-3p down-regulated. LncRNA GAS5 has been shown to bind to miR-375-3p by bioinformatic prediction. High expression of miR-375-3p has been found to be associated with cardiomyocyte hypertrophy and heart failure. Knock-down of MiR-375-3p suppresses cardiomyocyte hypertrophy induced by AngII (Feng et al., 2019). Garikipati et al. (2017) demonstrated that therapeutic inhibition of miR-375 attenuates inflammatory response and cardiomyocyte death, as well as enhancement of angiogenesis and cardiac function in a mouse myocardial infarction model.

Kruppel-like factor 4 (KLF4) plays an important role in cardiac hypertrophy. Cardiomyocyte-specific knockout of KLF4 aggravates ISO-induced cardiac hypertrophy, which suggests that control of KLF4 is a potential therapeutic target for cardiac hypertrophy (Yoshida et al., 2014). Based on bioinformatic prediction, *Klf4* is one of the target gene of miR-375-3p.

Hence, in this study, we hypothesized that lncRNA GAS5 sequesters miR-375-3p to promote KLF4 expression, mediating the anti-hypertrophic actions of OT. We attempted to identify gene transcription changes and their functions that respond to the treatment of oxytocin in cardiac hypertrophy. Our results will provide insights into discovery of potential therapeutic targets for cardiac hypertrophy.

## Materials and Methods

### Ethics Statement

In this study, all experimental procedures involving animals were approved by the Animal Care and Ethics Committee of Kunming Medical University (Kunming, China, Approval number: No. Kmmu2020350). All animal experiments complied with the National Institutes of Health Guide for the Care and Use of Laboratory animals. Significant efforts were made to minimize the sufferings and the number of the rats used.

### Experimental Animals and Drug Administration Protocol

**FIGURE 1:**
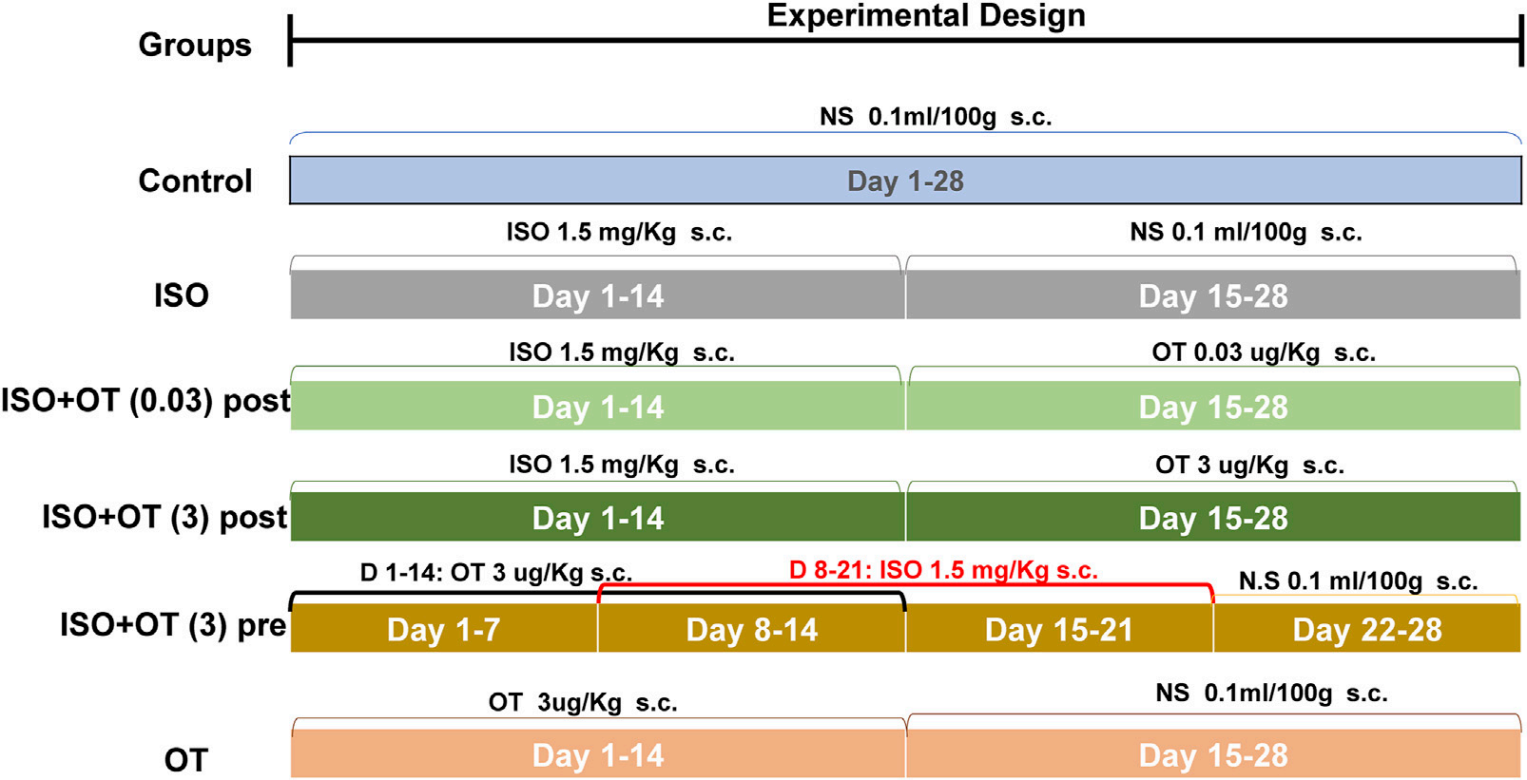
Illustration of the experimental protocols. NS, Normal Saline; ISO, Isoproterenol; OT, oxytocin; OTpost, oxytocin postconditioning; OTpre, oxytocin preconditioning; s.c, subcutaneous.

Male Sprague-Dawley (SD) rats weighing 220–250 g were purchased from Hunan SJA Laboratory Animal Co., Ltd., China. After 1 week acclimatization under laboratory conditions, a natural light cycle at a controlled temperature 25 ± 2°C with food and water available ad libitum, the rats were randomly divided into six groups (eight rats/group) and treated as Figure 1. In brief, the control group received a daily subcutaneous (s.c) injection of normal saline (0.1 ml/100 g) for 28 days. The ISO group received a daily s.c injection of isoproterenol (1.5 mg/kg/day) at day 1 to day 14, followed by a daily s.c injection of normal saline (0.1 ml/100 g) for another 14 days. The ISO+OT postconditioning group received a daily s.c injection of isoproterenol (1.5 mg/kg) for the first 14 days, followed by a daily s.c injection of 0.03 and 3 ug/kg oxytocin acetate (purchased from MedChemExpress) for the second 14 days, respectively. In ISO+OT preconditioning group, oxytocin acetate (3 ug/kg) was administered subcutaneously for 7 days, followed by co-administration of oxytocin and ISO for 7 days, and ISO administration alone for another 7 days. The time interval of administration between oxytocin acetate and ISO was more than 8 h. OT group received a daily s.c injection of oxytocin acetate (3 ug/kg) for the first 14 days, followed by a daily s.c injection of normal saline (0.1 ml/100 g) for the second 14 days. The dose of oxytocin acetate was based on Plante’s study (Plante et al., 2015). After 28-day treatment, echocardiography examinations were performed to assess changes in cardiac morphology and functions. At the end of the study, all animals were anesthetized using isoflurane in a sealed glass jar and hearts excised. Heart weights were measured after the hearts were rinsed with ice-cold saline and dried with filter papers. Some heart samples were immediately stored in liquid nitrogen, while the other samples were fixed in 4% paraformaldehyde.

### Echocardiography

Transthoracic echocardiograms were performed on rats using a PHILIPS EPIQ7C ultrasound system for cardiology. Rats were isolated in a glass chamber, anesthetized by inhalation of 1.5 to 2% isoflurane mixed with 100% oxygen, and then supinely placed on an operational warming platform. The chest hair was depilated with a hair removal cream. M-mode cine loops were recorded and analyzed by high-frequency ultrasound imaging software to assess myocardial parameters and cardiac functions of left ventricle (LV), including left ventricular posterior wall thickness at end-diastole (LVPWd), left ventricular posterior wall thickness at end-systole (LVPWs), left ventricular internal diameter at end-diastole (LVIDd), left ventricular internal diameter at end-systole (LVIDs), left ventricular ejection fraction (LVEF), and left ventricular fractional shortening (LVFS).

### Histomorphology Analysis

Heart tissues were fixed in 4% paraformaldehyde for 48 h and then embedded in paraffin. The embedded hearts were cut into 5-μm thickness sections, which were mounted on glass slide and stained with hematoxylin-eosin (H&E) and Masson’s trichrome stainings. The sections were assessed under light microscopic fields by digital image analysis (BH-Z, Olympus Corporation, Tokyo, Japan).

The tissue sections underwent dewaxing and rehydrated, then be boiled in EDTA buffer for 10 min, and phosphate-buffered saline was used to washing the slides three times. Slides were incubated for 30 min with 5 uM wheat germ agglutinin (WGA) dying (sigma) in the dark. The cell nucleus were stained with 4′,6-diamidino-2-phenylindole (DAPI). The slides were sealed with antifade mounting medium and then observed using a fluorescence microscope (Olympus BX53).

Masson’s trichrome staining was used to evaluate the myocardial fibrosis and WGA staining was used to evaluate the cardiomyocyte cross-sectional area. Myocardial fibrosis and cardiomyocyte cross-section areas were quantitatively analyzed with Image J software. Three images for each group and 40 cells for each image were randomly selected to determine the cross-section areas of cardiomyocyte. Five images for each group were randomly selected to measure the collagen content, which was presented by collagen volume fraction (collagen area/field area × 100%).

### Cell Culture and Treatment

Neonatal rat cardiomyocytes (NRCMs) were prepared from the hearts of 1- to 3-day-old SD rats. In brief, the hearts were excised from neonatal rats and washed in ice-cold phosphate buffered saline (PBS). Then the ventricles were finely separated and minced into 1–2 mm^3^ pieces, which were digested in 0.25% trypsin at 37°C. The cell suspension was centrifuged and the pellets re-suspended and then transferred into Dulbecco’s modified Eagle’s medium (DMEM) with 10% fetal bovine serum (FBS) and 1% penicillin/streptomycin (P/S) culture medium. Fibroblast growth inhibitor (sciencell) was added to the cell suspension. Immunofluorescence staining was performed to detect the expression of cardiac troponin T (cTnT) and immunofluorescence microscopy to identify primary cardiomyocytes. The cultured cardiomyocytes (CMs) were treated with vehicle alone, ISO (10 uM) alone or OT (10 nM) + ISO (10 uM) respectively and further incubated for 24 h before harvest. In OT + ISO group, the cells were pretreated with OT for 30 min prior to stimulation with ISO.

### Cell Transfection

LncRNA GAS5 and *Klf4* gene knock-down were achieved using RNA interference. shRNA-GAS5, shRNA- *Klf4* and its corresponding scramble negative control siRNA (siNC) were purchased from TsingKe Biological Technology (Beijing, China). miR-375-3p mimics, miR-375-3p inhibitor and the corresponding negative control were synthesized by RIBOBIO (Guangzhou, China). The *Klf4* over-expression plasmid (pcDNA-*Klf4*) and empty pcDNA3.1 plasmid (control) were purchased from RIBOBIO (Guangzhou, China).

Plasmids were transfected into the cultured CMs using Lipofectamine 3000 (Cat: L3000-015 Invitrogen) according to the manufacturer’s protocol. The transfection efficiency was detected by qRT-PCR and Western blotting. After 24-h transfection, the cells were incubated in DMEM medium supplemented with 10% FBS and 10 µM ISO for 24 h followed by 30 min OT pretreatment.

### Real-Time Quantitative Polymerase Chain Reaction

Total RNA was extracted from the cultured CMs and LV tissues using Trizol reagent (Lifetech 15596026). Reversely transcribed to complementary DNA (cDNA)for miR-375-3p and lncRNA GAS5 and PCR amplification were performed using a Bulge-Loop™ miRNA qRT-PCR Starter Kit, a Bulge-Loop™ miRNA qRT-PCR Primer Set and a lncDETECT™ lncRNA qRT-PCR kit (RiboBio, Guangzhou, China) according to the manufacturer’s instructions. U6 and β-actin served as internal normalized references for miR-375-3p and lncRNA GAS5, respectively. The relative mRNA levels of the target genes were evaluated by qRT-PCR with SYBR Green master mix (KAPA KK4601, Roche, United States) using the LightCycle 96 (Roche, United States). *In vitro* experiment, all qRT-PCR assays were conducted at least in triplicate. Fold-changes were calculated according to cycle quantitation (Ct) values with 2^−ΔΔCt^ method. List of the primers used for qRT-PCR is presented in Table 1.

**TABLE 1:** Primers for qRT-PCR.

Sequence (5′–3′)	Primer names
TAT​GGA​ATC​CTG​TGG​CAT​C	β-actin-F
GTG​TTG​GCA​TAG​AGG​TCT​T	β-actin-R
AATCTCACAGGCAGTTCT	GAS5-F
ATG​GCT​TTG​TTC​AGT​TAT​CC	GAS5-R
AAC​CTA​TAC​GAA​GAG​TTC​TCA​T	KLF4-F
CCAGTCACAGTGGTAAGG	KLF4-R
ACA​CTC​CAG​CTG​GGT​TTG​TTC​GTT​CGG​CTC	rno-miR-375-3p-F
CTC​AAC​TGG​TGT​CGT​GGA​GTC​GGC​AAT​TCA​GTT​GAG TCACGCGA	rno-miR-375-3p-R
GAT​GAT​TCT​GCT​CCT​GCT​TTT​CC	BNP-F
CAG​CTT​CTG​CAT​CGT​GGA​TT	BNP-R
CAG​CTC​AGT​CAT​GCC​AAC​CG	β-MHC-F
GCT​CCA​CGA​TGG​CGA​TGT​T	β-MHC -R
ACC​ATC​GGG​AAT​GAA​CGC​TT	α-SMA-F
CTG​TCA​GCA​ATG​CCT​GGG​TA	α-SMA-R
CTG​GAG​AAA​CCT​GCC​AAG​TAT​G	GAPDH-F
GGT​GGA​AGA​ATG​GGA​GTT​GCT	GAPDH-R

F, forward primer; R, reverse primer; qRT-PCR, real-time quantitative polymerase chain reaction.

### Western Blot Analysis

NRCMs or tissue samples were lysed by radioimmunoprecipitation assay (RIPA) lysis buffer (Beyotime Biotechnology, China). The total protein concentrations were determined by BCA protein assay kit (Beyotime Biotechnology, China). A total of 30 ug proteins were separated by 10% sodium dodecyl sulfate polyacrylamide gel electrophoresis (SDS-PAGE) and subsequently transferred onto polyvinylidene difluoride (PVDF) membranes. The blots were blocked with 5% non-fat milk and incubated overnight with the primary antibodies against BNP (1:500, rabbit #ab19645 abcam), β-MHC (1:1,000, rabbit #ab172967 abcam), KLF4 (1:1,000, rabbit #bs-1064R BIOSS), PI3K (1:1,000, rabbit #ab182651 abcam), p-PI3K (1:1,000, rabbit #205841-1-AP Proteintech), AKT1 (1:1,000, rabbit # bs-0115M BIOSS), p-AKT1 (1:1,000, rabbit # bs-0876R BIOSS), β-actin (1:1,000, rabbit #abmart P30002) and GAPDH (1:2,000, rabbit #2118S CST). Then, the membranes were washed and treated with horseradish peroxidase-conjugated secondary antibodies (CST 7074) at room temperature for 2 h. ECL System was employed to examine the protein bands and the intensity of protein bands measured by ImageJ software.

### Dual-Luciferase Reporter Gene Assay

LncRNA GAS5 or *Klf4* containing the predicted miR-375-3p binding sites were cloned into pGL3-WT-lncRNA Gas5, pGL3-MUT-lncRNA GAS5, pGL3-WT *Klf4* or pGL3-MUT *Klf4*, respectively. The WT or MUT reporter plasmid were co-transfected with the miR-375-3p mimics (50 nM) or the negative control into 293T cells using Lipofectamine 2000 (ThermoFisher), and the luciferase intensity was assessed by the double-luciferase reporter assay kit (Promega, United States) on **SpectraMax Gemini EM (Molecular Devices, United States).**

### Immunofluorescent Staining

After transfection and treatment, cultured cardiomyocytes were washed with cold PBS for three times and subsequently fixed with 4% paraformaldehyde for 30 min. After that, the cells were permeabilized with 3% Triton X-100 for 20 min. Then, washed three times with PBS, the cardiomyocytes were incubated with anti-cTnT antibody (1:300, rabbit bs-10648R) at 4°C overnight and were further incubated with fluorophore-conjugated secondary antibodies at a normal temperature for 1 h, and then stained with 4′,6-diamidino-2-phenylindole (DAPI) solution for 15 min. Immunofluorescence was analyzed under an inverted fluorescence microscope (Olympus BX53).

### Statistical Analysis

SPSS 20.0 (IBM Corp. Armonk, NY, United States) and Prism 8 (GraphPad Software, CA, United States) were used for statistical analysis and plotting. Data were expressed as mean ± standard deviation (sd). Unpaired Student’s *t*-test was used to determine the significance between two groups.

## Results

### Oxytocin Attenuated Hypertrophic Responses in ISO-Treated Cardiomyocytes

**FIGURE 2:**
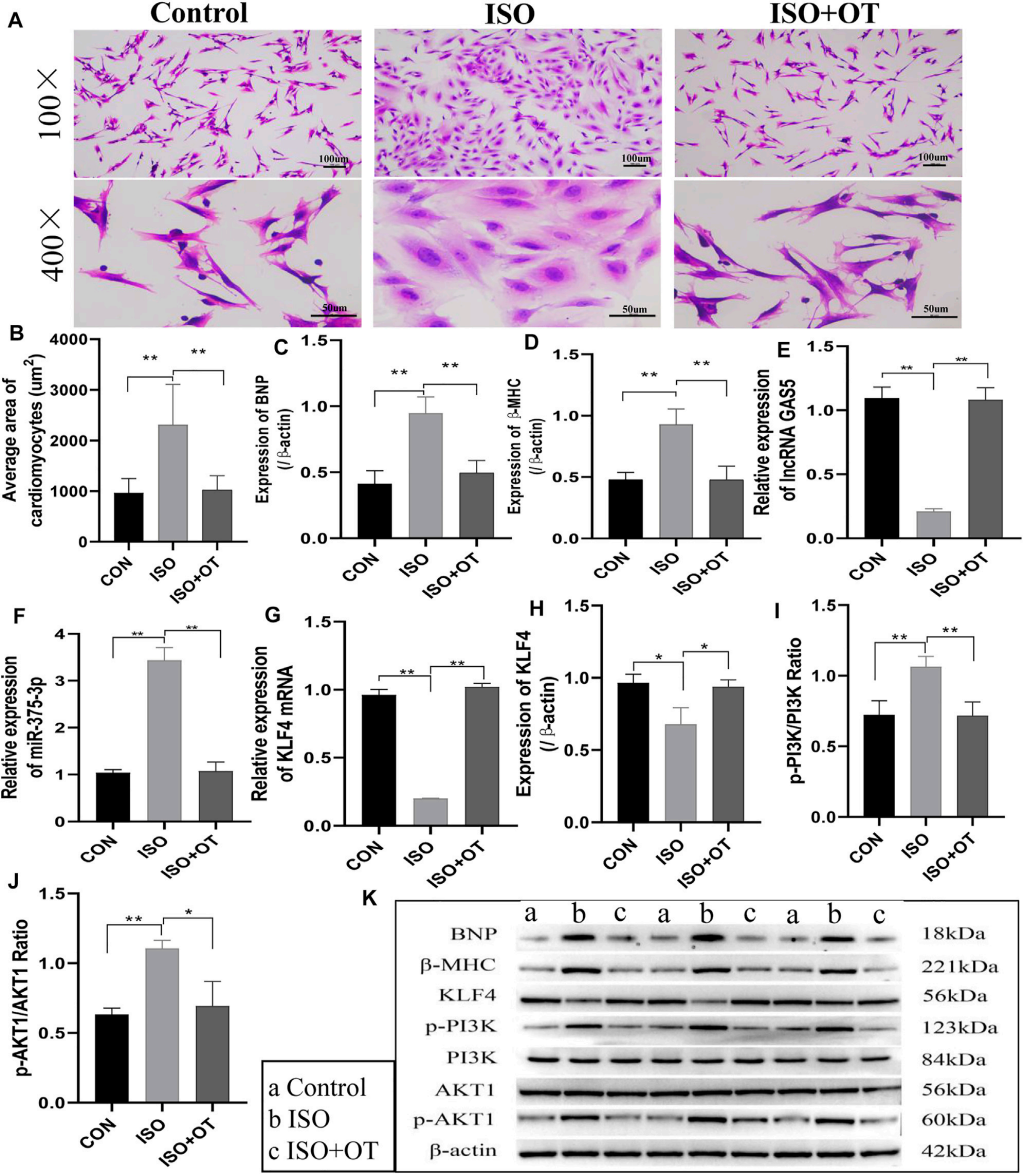
Antihypertrophic effects of OT *in vitro*. Neonatal rat cardiomyocytes stimulated with ISO for 24 h in the presence or absence of OT. **(A)** The cell morphology was evaluated by H&E staining. **(B)** Statistical results of measurement of cell surface areas. **(C,D)** Effects of OT on the protein expressions of BNP and β-MHC. **(E)** Effects of OT on the expression of lncRNA GAS5. **(F)** Effects of OT on the expression of miR-375-3p. **(G,H)** Effects of OT on the mRNA and protein expressions of KLF4. **(I)** Effects of OT on the p-PI3K/PI3K ratio. **(J)** Effects of OT on the p-AKT1/AKT1 ratio. **(K)** Western blot images of BNP, β-MHC, KLF4, p-PI3K, PI3K, AKT1, p-AKT1, and β-actin levels. Data are shown as the mean ± sd of three independent experiments. *, *p* < 0.05; **, *p* < 0.01.

First, to investigate the effects of OT pretreatment on cardiac hypertrophy, NRCMs were incubated and pretreated with OT for 30 min and then stimulated with 10 uM ISO for 24 h. Cardiomyocyte hypertrophy was evaluated by cell surface area and expressions of BNP and β-MHC, which have been well-established as cardiac hypertrophy markers. As shown in Figure 2, ISO triggered significant hypertrophic responses in NRCMs, as indicated by the increased cell surface area (Figures 2A,B) and elevated protein levels of BNP and β-MHC (Figures 2C,D,K), which were reduced by pretreatment of OT. At the same time, we observed the expressions of lncRNA GAS5 (Figure 2E), *Klf4* mRNA (Figure 2G) and KLF4 protein (Figures 2H,K) were down-regulated in ISO group when compared with control group, while up-regulated significantly after OT treatment. Inversely, the expression of miR-375-3p (Figure 2F) was increased significantly in ISO group when compared with control group and decreased after OT treatment. We also observed that OT modulated PI3K/AKT signaling pathway in a cardiomyocyte hypertrophy model. As shown in Figures 2I−K, the ratios of phosphorylated-PI3K/PI3K (p-PI3K/PI3K) and phosphorylated-AKT1/AKT1 (p-AKT1/AKT1) were significantly increased in ISO-induced hypertrophic cardiomyocytes and significantly decreased after OT treatment.

These results indicated that oxytocin could attenuate cardiomyocyte hypertrophic responses, promote the expressions of lncRNA GAS5 and KLF4 protein, repress miR-375-3p expression, and modulate PI3K/AKT signaling pathway.

### Oxytocin Protected Rats From ISO-Induced Cardiac Hypertrophy and Up-Regulated lncRNA GAS5 Expressions

**FIGURE 3:**
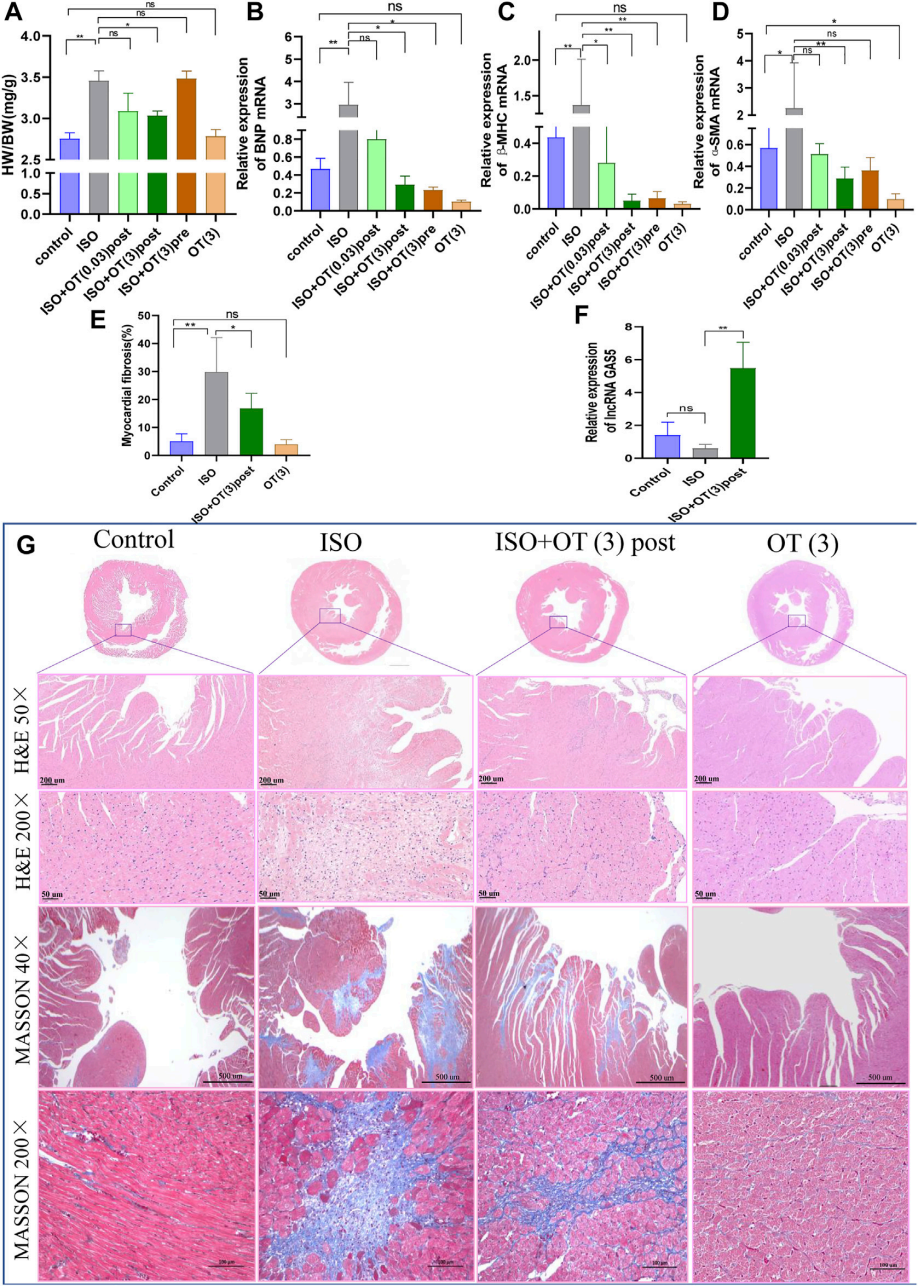
OT alleviated cardiac hypertrophy caused by ISO administration along with up-regulation of lncRNA GAS5 expression. SD rats were treated with ISO and different concentrations of OT as described. **(A)** HW/BW ratio. **(B−D)** The expressions of BNP, β-MHC and α-SMA mRNA detected by qRT-PCR. **(E)** Quantitative morphometric analysis of Masson’s trichrome staining representing myocardial fibrosis. **(F)** Effects of OT (3 ug/kg) postconditioning on the expression of lncRNA GAS5. **(G)** Representative histopathological images of heart tissue sections stained with H&E, Masson’s Trichrome staining from the indicated groups. *n* = 3–6 rats per experimental group. Data shown as mean ± sd, *p < 0.05 **p < 0.01.

To evaluate the effects of OT on cardiac hypertrophy *in vivo*, we established a rat cardiac hypertrophy model by ISO subcutaneous infusion for 14 days and treated rats with different dosages of OT before (preconditioning) and after (postconditioning) ISO infusions. Heart weight/body weight (HW/BW) ratio served as a measurement of cardiac hypertrophy. HW/BW ratio was significantly increased in ISO group compared to control group, but significant lower in ISO+OT (3) post group than that in ISO group (Figure 3A). HW/BW ratio was not significantly different in ISO+OT (0.03) post group and in ISO+OT (3) pre group when compared with ISO group (Figure 3A). Furthermore, no significant difference in HW/BW ratio was found between control group and OT (3) group (Figure 3A). Besides, ISO markedly increased the expressions of pathological cardiac hypertrophy markers (BNP, β-MHC) and fibrosis marker (Alpha-smooth muscle actin, α-SMA) compared with control group, whereas different concentrations of OT preconditioning or postconditioning inhibited ISO-induced increases in BNP, β-MHC and α-SMA (Figures 3B–D). Likewise, only high dosage (3 ug/kg) of OT postconditioning could significantly reduce expressions of hypertrophic and fibrosis markers (Figures 3B–D). From HE staining (Figure 3G), we observed a large number of inflammatory cells and fibroblasts gathered around endocardium region in ISO group and OT treatment alleviated it. Masson’s trichrome staining (Figure 3G) showed a significant increase in collagen deposition in ISO-infusion group and reduced in OT (3 ug/kg) postconditioning group (Figures 3E,G). To elucidate the mechanisms underlying the anti-hypertrophic effects of OT, lncRNA GAS5 expressions were detected in left ventricular tissues of different groups. As shown in Figure 3F, lncRNA GAS5 expressions in hypertrophic heart tissues were decreased, while up-regulated significantly in rat hearts treated with OT.

**FIGURE 4:**
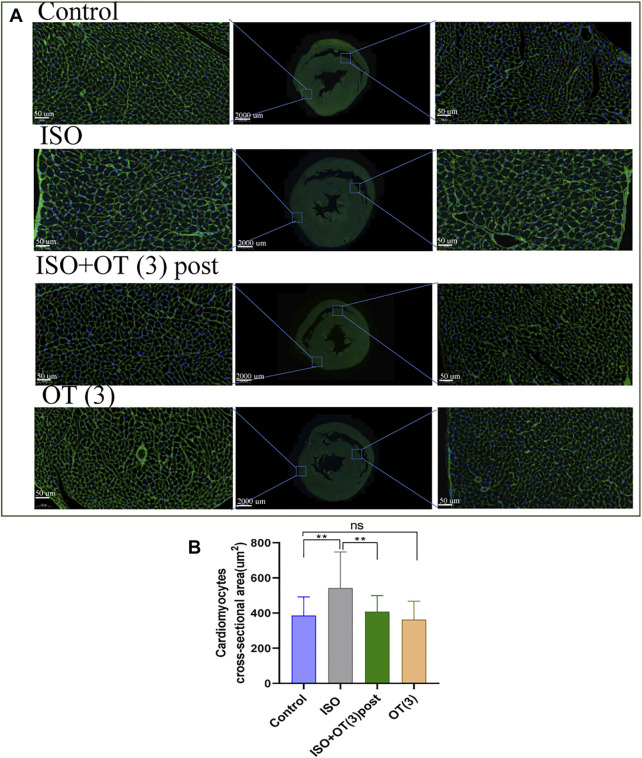
The cross sections of hearts were stained with WGA. **(A)** The representative images of WGA staining in the cardiomyocyte cross-sectional area. **(B)** Statistical results of measurement of cross-sectional areas of cardiomyocytes. Data are shown as mean ± sd, *p < 0.05 **p < 0.01.

The results of WGA staining (Figure 4A) showed that the cardiomyocytes were not orderly arranged and the cross-sectional areas of cardiomyocytes were significantly enlarged after ISO insulted, while OT (3 ug/kg) postconditioning could prevent these pathomorphological changes (Figure 4B).

### The Effects of OT on Cardiac Structure and Functional Parameters

**FIGURE 5:**
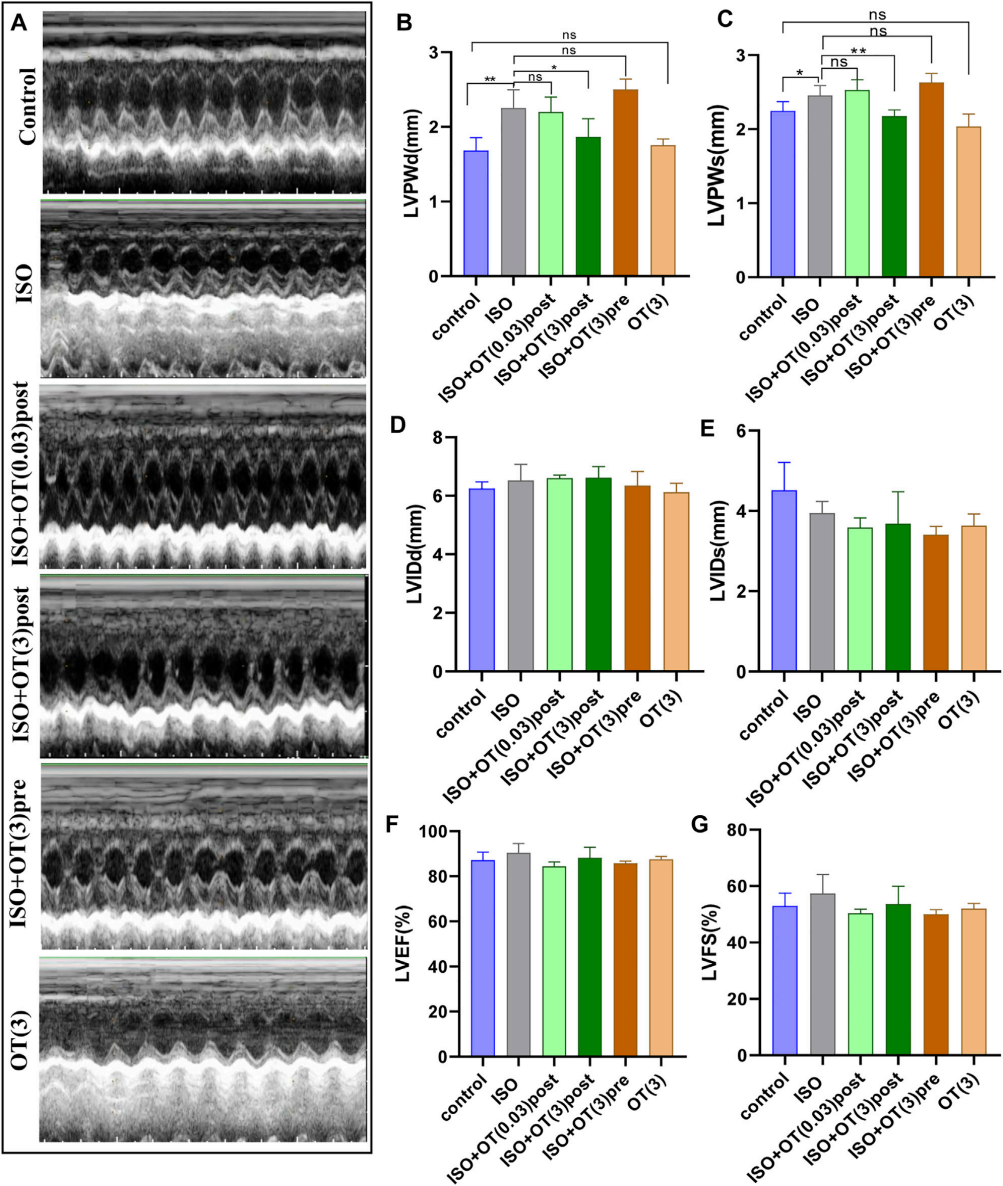
Effects of OT on cardiac structure and functional parameters assessed by Echocardiography. **(A)** Representative echo images of each group. **(B)** LVPWd. **(C)** LVPWs. **(D)** LVIDd. **(E)** LVIDs. **(F)** LVEF. **(G)** LVFS. *n* = 4–6 rats per experimental group. *p < 0.05; **p < 0.01; ns, not significant. LVPWd, left ventricular posterior wall thickness at end-diastole; LVPWs, left ventricular posterior wall thickness at end-systole; LVIDd, Left ventricular internal diameter end-diastole; LVIDs, Left ventricular internal diameter end-systole; LVEF, left ventricular ejection fraction; LVFS, left ventricular fractional shortening.

We performed transthoracic echocardiography to evaluate cardiac function and left ventricular remodeling (Figure 5A). ISO infusion induced cardiac hypertrophy of rats, as evidenced by increased LVPWd (Figure 5B) and LVPWs (Figure 5C) in ISO group compared with control group. OT (3 ug/kg) postconditioning treatment effectively prevented left ventricular wall from thickening (Figures 5B,C). There were no significant differences in LVIDd (Figure 5D), LVIDs (Figure 5E), LVEF (Figure 5F), and LVFS (Figure 5G) between ISO group and control group, suggesting that cardiac hypertrophy induced in our study was not typical concentric hypertrophy and left ventricular function was not worsened by ISO and not affected by OT.

### The Interactions of miR-375-3p With lncRNA GAS5 and *Klf4*

**FIGURE 6:**
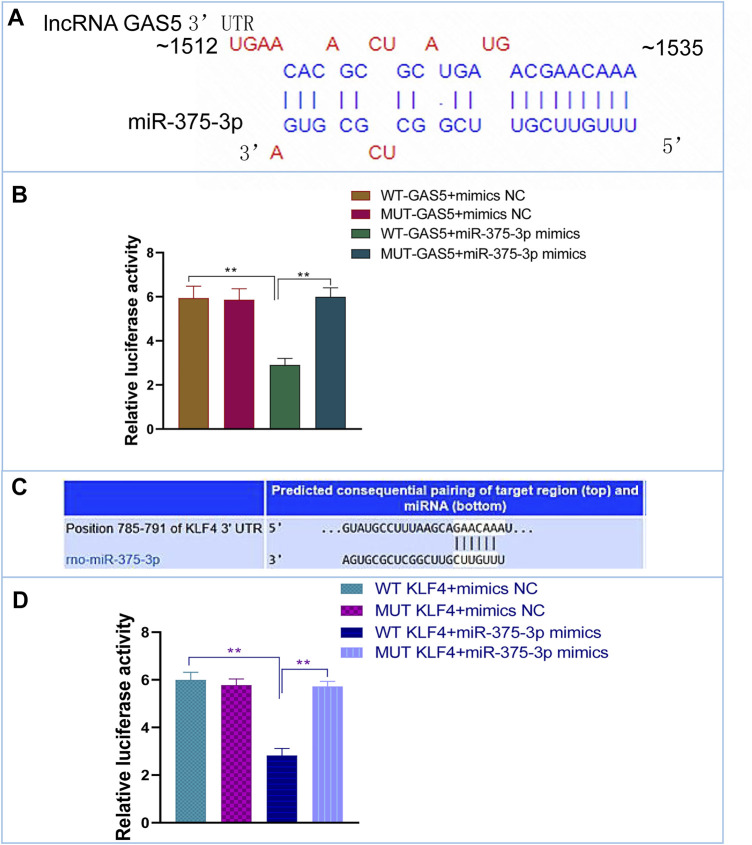
The interactions of miR-375-3p with lncRNA GAS5 and KLF4. **(A,C)** Predicted duplex formation between lncRNA GAS5 and miR-375-3p **(A)**, and KLF4 3′-UTR and miR-375-3p **(C)**. **(B)** The WT (MUT) GAS5 vector and miR-375-3p mimics or mimics NC were co-transfected into 293T cells. Dual luciferase gene reporter assay was performed to indicate that miR-375-3p could directly bind with the WT GAS5. **(D)** The WT (MUT) KLF4 3′-UTR vector and miR-375-3p mimics or mimics NC were co-transfected into 293T cells. Dual luciferase gene reporter assay was performed to indicate that miR-375-3p could directly bind with the WT KLF4. All the experiments were repeated three times. **p < 0.01. WT, wild-type; MUT, mutant type; NC, negative control.

Based on bioinformatic prediction (Figures 6A,C), we performed dual-luciferase reporter gene assays to validate the interactions between lncRNA GAS5 and miR-375-3p as well as miR-375-3p and *Klf4*. The results revealed that luciferase values were decreased in WT GAS5+miR-375-3p mimics group, but there was no significant alteration in MUT-GAS5+miR-375-3p mimics group or in mimics-NC+WT-GAS5/MUT-GAS5 groups (Figure 6B). Similarly, after co-transfection of WT *Klf4* and miR-375-3p mimics, the luciferase value was decreased significantly when compared with co-transfection of WT *Klf4* and miR-375-3p mimics NC group. There was no significant difference in luciferase value between MUT *Klf4*+mimics NC and MUT *Klf4*+miR-375-3p mimics groups (Figure 6D). These results confirmed that lncRNA GAS5 could sponge miR-375-3p and *Klf4* was a direct target gene of miR-375-3p.

### Knock-Down of lncRNA GAS5 Inhibited Anti-Hypertrophic Effects of Oxytocin via Upregulation of miR-375-3p

**FIGURE 7:**
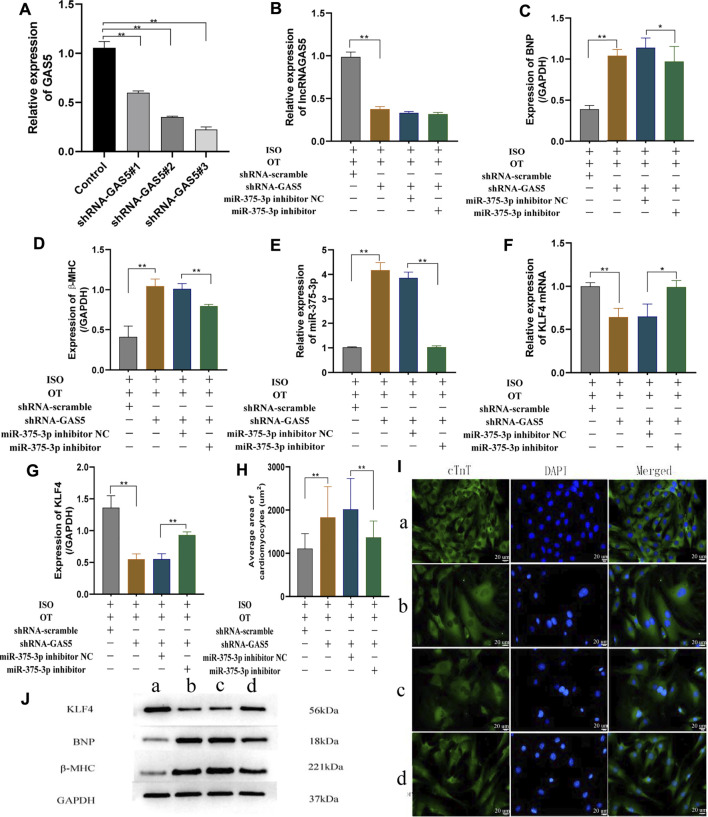
Down-regulation of lncRNA GAS5 inhibits the anti-hypertrophic effects of OT via miR-375-3p in ISO-treated primary cardiomyocytes. **(A)** qRT-PCR was performed to quantitatively measure lncRNA GAS5 expression after transfection of shRNA-GAS5#1, shRNA-GAS5#2, shRNA-GAS5#3 in cardiomyocytes. **(B)** Expression of lncRNA GAS5 was determined by qRT-PCR. **(C,D)** Expressions of BNP and β-MHC proteins were measured by Western blot analysis. **(E)** Expression of miR-375-3p was determined by qRT-PCR. **(F)** Expression of KLF4 mRNA was determined by qRT-PCR. **(G)** Expression of KLF4 protein was determined by Western blot analysis. **(H)** Statistical results of measurement of cell surface areas. **(I)** Cardiomyocyte surface areas were measured by immunofluorescent staining. Scale bars represent 20 µm. Images were captured at ×400 magnification **(J)** Representative Western blot images of KLF4, BNP, β-MHC and GAPDH. (a) ISO+OT+shRNA-scramble group. (b) ISO+OT+shRNA-GAS5 group. (c) ISO+OT+shRNA-GAS5+miR-375-3p inhibitor NC group. (d) ISO+OT+shRNA-GAS5+miR-375-3p inhibitor group. Data are shown as the mean ± sd of three independent experiments. *, *p* < 0.05; **, *p* < 0.01.

To validate the regulatory relationships among lncRNA GAS5, miR-375-3p and KLF4 in the *in vitro* model under OT action, NRCMs were transfected with shRNA GAS5 followed by OT pretreatment and ISO stimulation, and expressions of lncRNA GAS5, miR-375-3p and *Klf4* mRNA, as well as the protein levels of KLF4, BNP and β-MHC were evaluated. At the beginning, we assessed the transfection efficiency of shRNA GAS5#1, shRNA GAS5#2, shRNA GAS5#3 in cardiomyocytes and selected the shRNA GAS5#3 (shRNA-GAS5) for the subsequent experiments due to the highest efficiency (Figure 7A). The expression of lncRNA GAS5 was significantly decreased in ISO+OT+shRNA-GAS5 group compared with ISO+OT+shRNA-scramble group (Figure 7B), suggesting that knock-down of lncRNA GAS5 was effective and sustained. Compared to ISO+OT+shRNA-scramble group, the expressions of BNP, β-MHC were significantly increased in ISO+OT+shRNA-GAS5 group (Figures 7C,D,J). Meanwhile, we also found that after lncRNA GAS5 knock-down, the expression of miR-375-3p was increased (Figure 7E), while *Klf4* mRNA (Figure 7F) and protein (Figures 7G,J) levels were decreased. These results indicated that knock-down of lncRNA GAS5 inhibited anti-hypertrophic effects of oxytocin and that lncRNA GAS5 negatively regulated miR-375-3p and positively regulated *Klf4* mRNA and protein.

Subsequently, we co-transfected shRNA GAS5 and miR-375-3p inhibitor to NRCMs followed by OT and ISO treatment. The expression of miRNA-375-3p was markedly down-regulated in ISO+OT+shRNA-GAS5+miR-375-3p inhibitor group compared with ISO+OT+shRNA-GAS5+miR-375-3p inhibitor NC group (Figure 7E). The inhibiting effects of shRNA-GAS5 on *Klf4* mRNA and protein expressions were reversed by transfection of miR-375-3p inhibitor (Figures 7F,G,J). Meanwhile, the expressions of hypertrophic markers (BNP and β-MHC) (Figures 7C,D,J) as well as the size of the cardiomyocytes evaluated by immunofluorescent staining (Figures 7H,I) were significantly decreased in ISO+OT+shRNA-GAS5+miR-375-3p inhibitor group compared with ISO+OT+shRNA-GAS5+miR-375-3p inhibitor NC group. These results indicated that miR-375-3p inhibitor rescued the pro-hypertrophic effects of shRNA-GAS5.

Taken together, these data demonstrated that lncRNA GAS5 mediated anti-hypertrophic effects of OT through negative regulation of miR-375-3p expression.

### Upregulation of miR-375-3p Blunted Anti-Hypertrophic Effects of Oxytocin via Downregulation of KLF4 and Modulation of PI3K/AKT Signaling Pathway

**FIGURE 8:**
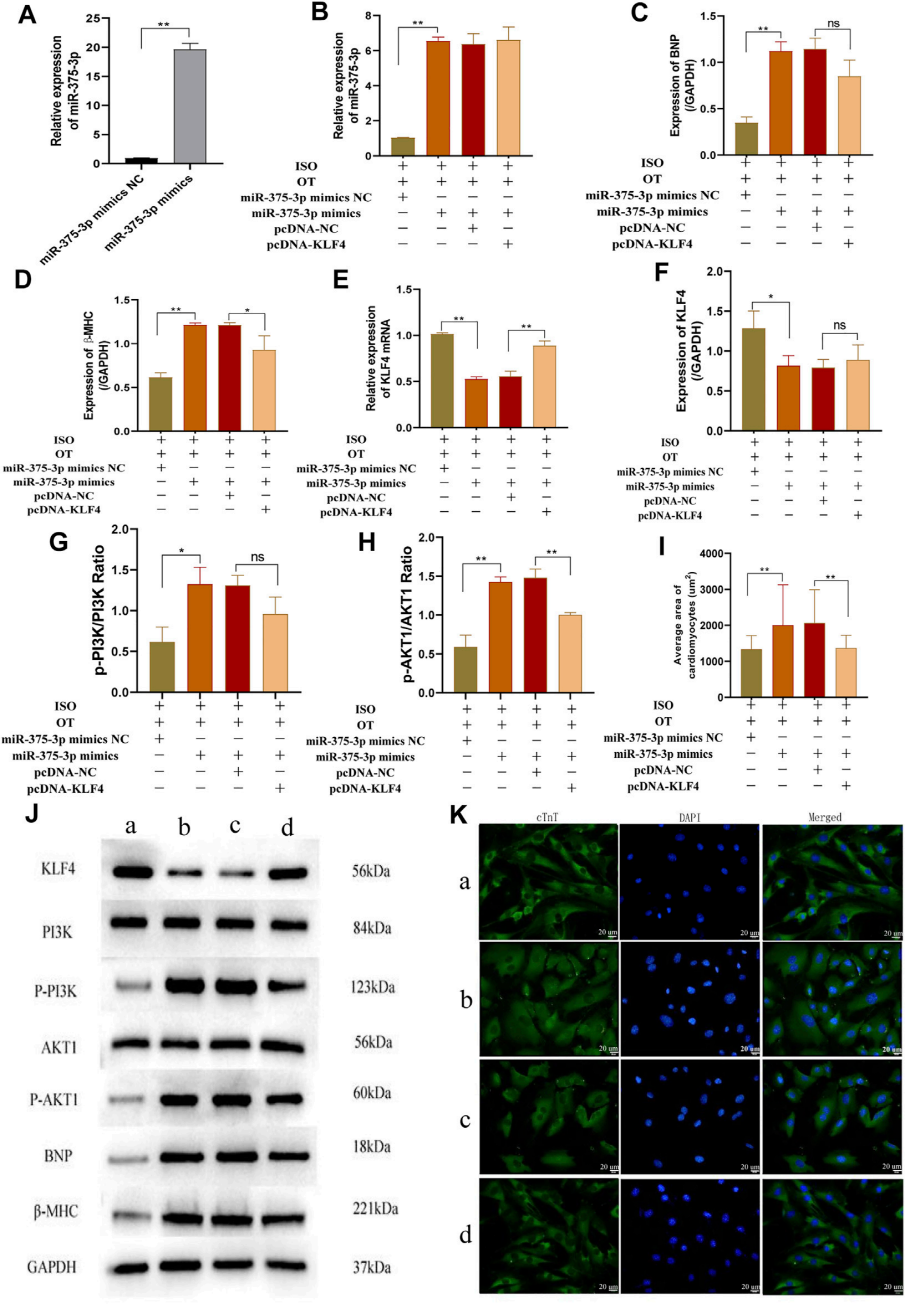
Over-expression of miR-375-3p blunted anti-hypertrophic effects of oxytocin via KLF4 and modulated the PI3K/AKT signaling pathway. **(A)** Expression of miR-375-3p in cardiomyocytes following transfection of miR-375-3p mimics and miR-375-3p mimics NC. **(B)** Expression of miR-375-3p was detected by qRT-PCR. **(C,D)** Expressions of BNP and β-MHC proteins were measured by Western blot analysis. **(E)** Expression of KLF4 mRNA was detected by qRT-PCR. **(F)** Expression of KLF4 protein was measured by Western blot analysis. **(G)** p-PI3K/PI3K ratio. **(H)** p-AKT1/AKT1 ratio. **(I)** Statistical results of measurement of cell surface areas. **(J)** Representative western blot images of KLF4, PI3K, p-PI3K, AKT1, p-AKT1, BNP, β-MHC and GAPDH levels. **(K)** Cardiomyocyte surface areas were measured by immunofluorescent staining. Scale bars represent 20 µm. Images were captured at ×400 magnification. (a) ISO+OT+miR-375-3p mimics NC group. (b) ISO+OT+miR-375-3p mimics group. (c) ISO+OT+miR-375-3p mimics+pcDNA-NC group. (d) ISO+OT+miR-375-3p mimics+pcDNA-KLF4 group. Data are shown as the mean ± sd of three independent experiments. *, *p* < 0.05; **, *p* < 0.01.

To further verify whether down-regulation of miR-375-3p mediated the anti-hypertrophic effects of OT, we upregulated miR-375-3p by transfection of miR-375-3p mimics into NRCMs followed by OT pretreatment and ISO insult. The transfection efficiency of miR-375-3p mimics in cardiomyocytes was detected and demonstrated to be effective (Figure 8A). Forced expression of miR-375-3p was observed in ISO+OT+miR-375-3p mimics group, but not in ISO+OT+miR-375-3p mimics NC group (Figure 8B). As compared with ISO+OT+miR-375-3p mimics NC group, ISO+OT+miR-375-3p mimics group presented notable increases in BNP and β-MHC expressions (Figures 8C,D,J), along with remarkable decreases in *Klf4* gene (Figure 8E) and protein (Figures 8F,J), suggesting that upregulation of miR-375-3p dampened anti-hypertrophic effects of OT and miR-375-3p could negatively regulate expressions of KLF4. Rescue assays were carried out to test whether upregulation of *Klf4* could abolish the effects conferred by miR-375-3p mimics. We co-transfected miR-375-3p mimics and pcDNA-*Klf4* vector or empty pcDNA vector into NRCMs followed by OT and ISO treatments. The results showed that the expression of *Klf4* mRNA was significantly increased in ISO+OT+miR-375-3p mimics+pcDNA-*Klf4* group compared to ISO+OT+miR-375-3p mimics+pcDNA-NC group (Figure 8E), whereas the expressions of KLF4 protein of two groups were not statistically significant (Figures 8F,J). β-MHC protein was reduced observably in ISO+OT+miR-375-3p mimics+pcDNA-*Klf4* group compared with ISO+OT+miR-375-3p mimics+pcDNA-NC group (Figures 8D,J), while BNP protein was not significantly reversed (Figures 8C,J). Immunofluorescent staining was also used to detect the changes in cardiomyocyte sizes (Figure 8K). Cardiomyocytes were markedly enlarged in ISO+OT+miR-375-3p mimics group compared with ISO+OT+miR-375-3p mimics NC group. Compared with ISO+OT+miR-375-3p mimics+pcDNA NC group, the sizes of cardiomyocytes were diminished notably in ISO+OT+miR-375-3p mimics+pcDNA-*Klf4* group (Figures 8I,K).

Furthermore, we also evaluated the protein expressions of p-PI3K, PI3K, p-AKT1, AKT1 after transfection of miR-375-3p mimics and co-transfection of miR-375-3p mimics and pcDNA-*Klf4*, respectively. The ratios of p-PI3K/PI3K (Figures 8G,J) and p-AKT1/AKT1 (Figures 8H,J) were significantly increased in ISO+OT+miR-375-3p mimics group compared with ISO+OT+miR-375-3p mimics NC group, suggesting that PI3K/AKT signaling pathway was also regulated by miR-375-3p. When the cells were simultaneously transfected with miR-375-3p mimics and pcDNA-*Klf4*, the ratio of p-AKT1/AKT1 (Figures 8H,J) was significantly reduced, whereas p-PI3K/PI3K (Figures 8G,J) was reduced but not statistically significant.

### *Klf4* Knock-Down Inhibited Anti-Hypertrophic Effects of Oxytocin via PI3K/AKT Pathway

**FIGURE 9:**
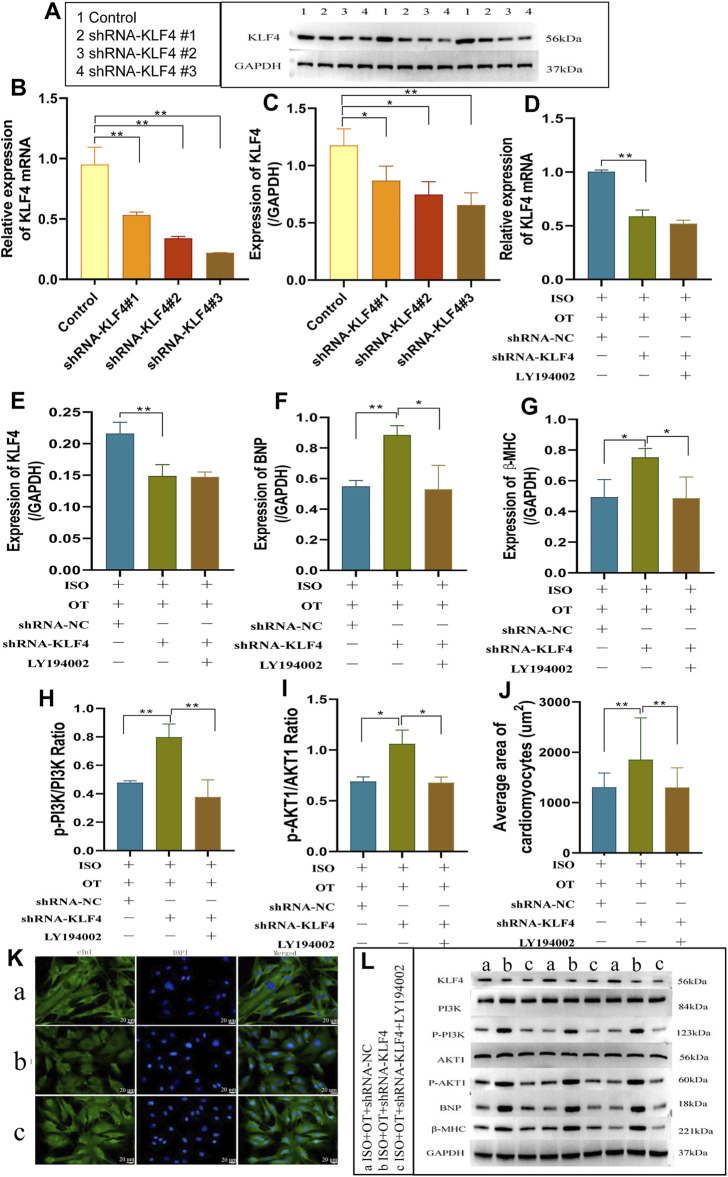
knock-down of KLF4 blunted anti-hypertrophic effects of oxytocin via PI3K/AKT pathway. **(A–C)** Detection of relative shRNA-KLF4 interference effects. The changes in KLF4 mRNA and protein levels were detected by qRT-PCR and Western blotting after transfection of shRNA-KLF4 #1, shRNA-KLF4 #2, shRNA-KLF4 #3 in primary cardiomyocytes. **(D)** Expression of KLF4 mRNA was determined by qRT-PCR. **(E)** Expression of KLF4 protein was measured by Western blot analysis. **(F,G)** Expressions of BNP, β-MHC proteins were measured by Western blot analysis. **(H)** p-PI3K/PI3K ratio. **(I)** p-AKT1/AKT1 ratio. **(J)** Statistical results of measurement of cell surface areas. **(K)** Cardiomyocyte surface areas were measured by immunofluorescent staining. Scale bars represent 20 µm. Images were captured at ×400 magnification. **(L)** Western blot images of KLF4, PI3K, p-PI3K, AKT1, p-AKT1, BNP, β-MHC and GAPDH levels. (a) ISO+OT+shRNA-NC group. (b) ISO+OT+shRNA-KLF4 group. (c) ISO+OT+shRNA-KLF4+LY194002 group. Data are shown as the mean ± sd of three independent experiments. *, *p* < 0.05; **, *p* < 0.01.

We first assessed the interference efficiency of shRNA *Klf4*#1, shRNA *Klf4*#2, and shRNA *Klf4*#3 in cardiomyocytes and then selected the shRNA *Klf4*#3 for the subsequent experiments based on its highest efficiency of interference (Figures 9A–C). We transfected shRNA-*Klf4* #3 (shRNA-*Klf4*) and its negative scramble into NRCMs followed by OT pretreatment and ISO stimulation. The expressions of *Klf4* mRNA and protein were significantly lower in ISO+OT+shRNA-*Klf4* group than those in ISO+OT+shRNA-NC group (Figures 9D,E,L). The anti-hypertrophic effects of OT was dampened in ISO+OT+shRNA-*Klf4* group compared with ISO+OT+shRNA-NC group, as shown by higher protein expressions of BNP and β-MHC (Figures 9F,G,L), and larger surface areas of cardiomyocytes observed in ISO+OT+shRNA-*Klf4* group (Figures 9J,K).

The ratios of p-PI3K/PI3K (Figures 9H,L) and p-AKT1/AKT1 (Figures 9I,L) in ISO+OT+shRNA-*Klf4* group were significantly higher than those in ISO+OT+shRNA-NC group, suggesting PI3K/AKT pathway was modulated by KLF4. After transfecting shRNA-*Klf4* into NRCMs, we pretreated NRCMs with 10 uM LY194002 (a PI3K inhibitor that prevents PI3K phosphorylation) and then treated with OT and ISO. The results revealed that the ratios of p-PI3K/PI3K (Figures 9H,L) and p-AKT1/AKT1 (Figures 9I,L) were notably lower in ISO+OT+shRNA-*Klf4*+LY194002 group than those in ISO+OT+shRNA-*Klf4* group. The expressions of BNP and β-MHC (Figures 9F,G,L) as well as the sizes of cardiomyocytes were remarkably reduced in ISO+OT+shRNA-*Klf4*+LY194002 group compared with ISO+OT+shRNA-*Klf4* group (Figures 9J,K). These findings suggested that blockade of PI3K/AKT signaling pathway rescued the pro-hypertrophic effects of shRNA- *Klf4*.

The above results indicated that KLF4 mediated the anti-hypertrophic effects of OT, and PI3K/AKT is the downstream signaling pathway of miR-375-3p/KLF4 axis.

## Discussion

Oxytocin has a broad spectrum of beneficial roles in regulating cardiovascular homeostasis (Reiss et al., 2019; Jankowski et al., 2020). An *ex vivo* experiment well documented that OT treatment exerts anti-hypertrophic responses in cardiomyocytes exposed to endothelin-1 and AngII (Menaouar et al., 2014). We constructed cardiomyocyte hypertrophic model by ISO challenge and found that OT exerted cardioprotection against hypertrophy, which is consistent with a previous report (Menaouar et al., 2014). In an *in vivo* study, Plante et al. (2015) reported that chronic subcutaneous infusion OT prevents the development of cardiomyocyte hypertrophy, fibrosis associated with obesity and diabetes in db/db mice. Similarly, Garrott et al. (2017) showed that chronic activation of hypothalamic oxytocin neurons blunts cellular hypertrophy and myocardial collagen density in rats undergoing *trans*-ascending aortic constriction through increase of parasympathetic tone. Our *in vivo* findings are in accordance with aforementioned studies (Plante et al., 2015; Garrott et al., 2017). In contrast, a recent study demonstrated that OT accelerated AngII-induced cardiac hypertrophy and fibrosis when the rats were simultaneously infused AngII and OT for 28 days (Phie et al., 2015). This discrepancy over anti-hypertrophic effects of OT *in vivo* studies may be attributed to the different administration routes (central or peripheral) or different concentrations of OT.

Our study showed that different doses of OT preconditioning or postconditioning inhibited hypertrophic responses induced by ISO and that OT (3 ug/kg) postconditioning exhibited more significantly anti-hypertrophic and anti-fibrotic effects. The delivery route, dose, and timing of OT possibly affected therapeutic effects. In our preliminary experiment, we initially investigated the actions of OT preconditioning, in spite of that the infusion time interval of OT and ISO was more than 2 h, the mortality of rats receiving these two agents was still high. Accordingly, we adjusted the infusion time interval to more than 8 h and the survival rate of rats was improved in preconditioning group. Oxytocin is a vasoactive hormone, which could induce both vasoconstriction or vasodilation depending on doses used, and decrease the LV preload and the inotropic state (Carter et al., 2020). Phie et al. (2015) documented that OT elicited a detrimental effect when AngII was simultaneously infused. The possible reasons may be due to that the rats hearts undergo complex effects caused by disturbances of parasympathetic and sympathetic system in response to AngII and OT, some of which are harmful rather than cardioprotective, and therefore counteract the beneficial effects of OT.

Oxytocin has been well documented to be implicated in stress-buffering via regulation of autonomic nervous system (Tracy et al., 2018; Carter et al., 2020; Belém-Filho et al., 2021). It is well known that chronic stresses, including social, emotional, and physical stresses, increase activity of sympathetic nervous system. Recently, Belém-Filho et al. (2021) reported that OT attenuates tachycardiac responses evoked by restraint via increasing parasympathetic activity, promoting cardioprotection by reducing the stress-evoked heart rate increase. Jovanovic et al. (2019) provided evidence that oxytocin treatment decreases the heart/body weight ratio and prevents the hypertrophy of cardiomyocytes in the wall of the left ventricle of the stressed rats. The present study showed that the expression levels of hypertrophy and fibrosis markers in OT alone group were lower than those in control group, which may be associated with the anxiolytic effects of Oxytocin, because daily infusion of normal saline or OT for 28 days was a kind of chronic stressor for rats. The underlying mechanisms by which OT regulates cardiac hypertrophy remain largely unknown. Over the past decade, some non-coding RNAs have been identified to mediate the development of cardiac hypertrophy, whereas the roles of non-coding RNAs in OT-evoked antihypertrophic effects are not well established. In our study, we identified oxytocin modulated lncRNA GAS5 and miR-375-3p expressions in the hypertrophic cardiomyocytes, and validated that *Klf4* is a direct target gene of miR-375-3p. lncRNA GAS5 is a potential regulator of hypertension-related vascular remodeling: Downregulation of lncRNA GAS5 was observed in spontaneously hypertensive rats, which had elevated blood pressure and increased medial thickness and luminal diameter (Wang et al., 2016). LncRNA GAS5 expression is downregulated in cardiomyocytes in pathological cardiac hypertrophy induced by ISO (Liu et al., 2019) and in diabetic cardiomyopathy (Xu et al., 2020a). Therefore up-regulation of lncRNA GAS5 attenuates cardiac fibrosis, myocardial hypertrophy and improved cardiac function (Liu et al., 2019; Xu et al., 2020a). The present study showed that anti-hypertrophic effects of OT were accompanied by up-regulation of lncRNA GAS5 and that downregulation of lncRNA GAS5 minimized anti-hypertrophic effects of OT. These results were in line with previous studies (Wang et al., 2016; Liu et al., 2019; Xu et al., 2020a).

MiR-375-3p was recently found to promote cardiac hypertrophy by reducing protein expression of lactate dehydrogenase B chain, a regulator of cell metabolism (Feng et al., 2019). In addition, miR-375 is significantly up-regulated in post-myocardial infarction mice hearts (Garikipati et al., 2015) and failing human hearts (Garikipati et al., 2017). Therapeutic inhibition of miR-375 decreases inflammatory response, reduces cardiomyocyte apoptosis in ischemic myocardia, and attenuates LV remodeling after myocardial infarction (Garikipati et al., 2017). KLF4, a novel anti-hypertrophic transcriptional regulator, inhibits cardiomyocyte hypertrophy induced by phenylephrine in cultured cardiomyocytes or by partial aortic constriction in mice (Kee and Kook, 2009; Liao et al., 2010). It has been documented that KLF4 mediates anti-hypertrophic effects of histone deacetylase inhibitors (Kee and Kook, 2009), berberine (Ding et al., 2021), and lncRNA-Mhrt (Xu et al., 2020b). In this study, miR-375-3p was up-regulated in hypertrophic cardiomyocytes, but downregulated by OT treatment. The expressions of *Klf4* mRNA and protein were notably decreased in hypertrophic cardiomyocytes but increased after OT treatment. We hypothesized lncRNA GAS5/miR-375-3p/KLF4 axis mediated the anti-hypertrophic actions of OT. We transfected shRNA-GAS5, miR-375-3p mimics and shRNA-*Klf4* followed by OT treatment and ISO stimulation respectively to evaluate the changes in anti-hypertrophic effects of OT. Our data showed that shRNA-GAS5, shRNA-*Klf4*, and miR-375-3p mimics could minimize anti-hypertrophic effects of OT. Meanwhile, after lncRNA GAS5 knock-down, miR-375-3p was up-regulated and KLF4 levels significantly downregulated. Over-expression of miR-375-3p resulted in inhibition of KLF4 expression. Through dual-luciferase reporter gene assays, we confirmed the regulatory relationship between lncRNA GAS5 and miR-375-3p, as well as miR-375-3p and *Klf4*. Additionally, we conducted the rescue experiments to further confirm the functions of lncRNA GAS5, miR-375-3p, and KLF4, respectively. lncRNA GAS5 knock-down-induced hypertrophy was reversed after co-transfection with miR-375-3p inhibitor. Similarly, miR-375-3p over-expression increased expression of hypertrophic fetal genes, which was partially reversed after co-transfection with *Klf4* pcDNA. To sum up, our data elaborate the regulatory relationships among lncRNA GAS5, miR-375-3p and KLF4, which imply that lncRNA GAS5 functions as a decoy of miR-375-3p, leads to enhanced KLF4 expression, and mediates anti-hypertrophic effects of OT.

From Figure 8E, we confirmed the pcDNA-*Klf4* transfection efficiency, as the expression of *Klf4* mRNA is significantly increased in ISO+OT+miR-375-3p mimics+pcDNA-*Klf4* group compared to ISO+OT+miR-375-3p mimics+pcDNA-NC group. However, the expressions of KLF4 protein showed a slightly but not significantly increased in ISO+OT+miR-375-3p mimics+pcDNA-*Klf4* group compared with ISO+OT+miR-375-3p mimics+pcDNA-NC group (Figure 8F). The reason can be explained by the presence of miR-375-3p mimics. We co-transfected miR-375-3p mimics and pcDNA-*Klf4* into the cardiomyocytes, due to *Klf4* mRNA is the direct target gene of miR-375-3p, miR-375-3p mimics mediated the post-transcriptional silence effects on *Klf4* mRNA, so KLF4 protein expressions were repressed. Due to the insufficient expressions of KLF4 proteins, the downstream signaling pathway was affected. The p-PI3K/PI3K ratio (Figure 8G) was not significantly decreased as expected in ISO+OT+miR-375-3p mimics+pcDNA-*Klf4* group than in ISO+OT+miR-375-3p mimics+pcDNA-NC group. Similarly, the rescue effects of up-regulation of KLF4 on inhibiting hypertrophic markers, the BNP expression (Figure 8C), was not significant.

It is well established that PI3K/AKT signaling pathway is a key pathway in the process of cardiac hypertrophy. Excessive activation of PI3K/AKT cascade contributes to the progress of cardiac hypertrophy, which has been observed in cardiac-selective transgenic over-expression of AKT in mice (Condorelli et al., 2002) and other cardiac hypertrophy models (Zhu et al., 2009; Gao et al., 2017). Effectively suppressing PI3K/AKT signaling by pharmacological agents could be resistant to cardiac hypertrophy when mouse hearts are exposed to chronic pressure overload, as evidenced by that Isorhamnetin (Gao et al., 2017) and Astragaloside IV (Liu et al., 2018) protect against cardiac hypertrophy through inactivation of PI3K/AKT pathway. Consistently, we found the expressions of p-PI3K and p-AKT were increased by ISO treatment, while significantly decreased by OT treatment. PI3K/AKT signaling pathway has been proposed to be implicated in the cardioprotective effect of OT by several studies: OT increases myocardial glucose uptake via triggering PI3K phosphorylation (Florian et al., 2010); OT protects H9c2 cells against I/R by recruiting p-AKT accumulated in the perinuclear region (Gonzalez-Reyes et al., 2015). Both of these cardioprotective effects exerted by OT were abrogated by PI3K inhibitor Wortmannin, which suggests that PI3K/AKT signaling pathway, a salvage kinase pathway, is responsible for cardioprotection of OT (Florian et al., 2010; Gonzalez-Reyes et al., 2015).

PI3K/AKT was shown to be a downstream signaling pathway of KLF4 in the study of carcinoma (Chang et al., 2015; Tang et al., 2018). Therefore, we further determined whether PI3K/AKT pathway is regulated by miR-375-3p/KLF4 axis under OT treatment in the *in vitro* hypertrophic model. We checked the expressions of PI3K/AKT pathway-related proteins after miR-375-3p over-expression and *Klf4* knock-down, respectively, and found that either miR-375-3p over-expression or *Klf4* knock-down significantly up-regulated phosphorylated PI3K and AKT in cultured cardiomyocytes. Furthermore, LY194002, an inhibitor of PI3K, abrogated sh-*Klf4*-evoked cardiac hypertrophy. These results indicate that inactivation of PI3K/AKT signaling by OT may therefore play a causal role in its anti-hypertrophic action.

## Limitations

In our *in vivo* study, we showed the anti-hypertrophic effects of exogenous oxytocin administration on ISO-induced cardiac hypertrophy. Since oxytocin is an endogenic hormone and oxytocin and oxytocin receptors are located in the heart, oxytocin concentrations in hypertrophic heart tissues should have been assayed. Moreover, a lack of evaluation of cardiac diastolic function of rats is the limitation of this study. Our study revealed the role of lncRNA GAS5 and KLF4 in cardiac hypertrophy in an *in vitro* model, not in an *in vivo* one. Therefore, much more work needs to be done to validate lncRNA GAS5 and KLF4 as potential mediating factors in the anti-hypertrophic effects of OT in an *in vivo* model.

## Conclusion

Taken together, OT-conferred anti-hypertrophic effects are mediated via inhibiting PI3K/AKT signaling pathway through up-regulating lncRNA GAS5 and KLF4 and down-regulating miR-375-3p. Our findings provide not only new evidence that OT could be an agent to ameliorate cardiac hypertrophy, but also insights into the potential therapeutic targets for cardiac hypertrophy.

## Data Availability

The original contributions presented in the study are included in the article/Supplementary Material, further inquiries can be directed to the corresponding author.

## Abbreviations

OT, oxytocin; ISO, isoproterenol; microRNA, miRNA; lncRNA, long non-coding RNA; GAS5, Growth Arrest-Specific transcript 5; KLF4, Kruppel like factor 4; PI3K, phosphatidylinositol 3-kinase; AKT, Protein Kinase B; BNP, B-Natriuretic Peptide; β-MHC, β-myosin heavy chain; HF, heart failure; AngII, angiotensin II; OXTR, oxytocin receptor; I/R, ischemia-reperfusion; ncRNAs, non-coding RNAs; H&E, hematoxylin-eosin; NRCMs, Neonatal rat cardiomyocytes; CMs, Cultured cardiomyocytes; LV, left ventricular; LVPWd, left ventricular posterior wall thickness at end-diastole; LVPWs, left ventricular posterior wall thickness at end-systole; LVIDd, left ventricular internal diameter at end-diastole; LVIDs, left ventricular internal diameter at end-systole; LVEF, left ventricular ejection fraction; LVFS, left ventricular fractional shortening; WGA, wheat germ agglutinin.
