# Supplementary material for: Oxytocin Protects Against Isoproterenol-Induced Cardiac Hypertrophy by Inhibiting PI3K/AKT Pathway via a lncRNA GAS5/miR-375-3p/KLF4-Dependent Mechanism
Source: Front Pharmacol. 2021 Dec 3;12:766024. doi: 10.3389/fphar.2021.766024 (PMC8678504; doi:10.3389/fphar.2021.766024)
Supplement: Supplementary file 1 [file DataSheet1.PDF]

Figure 2. Original western blot for three repeats

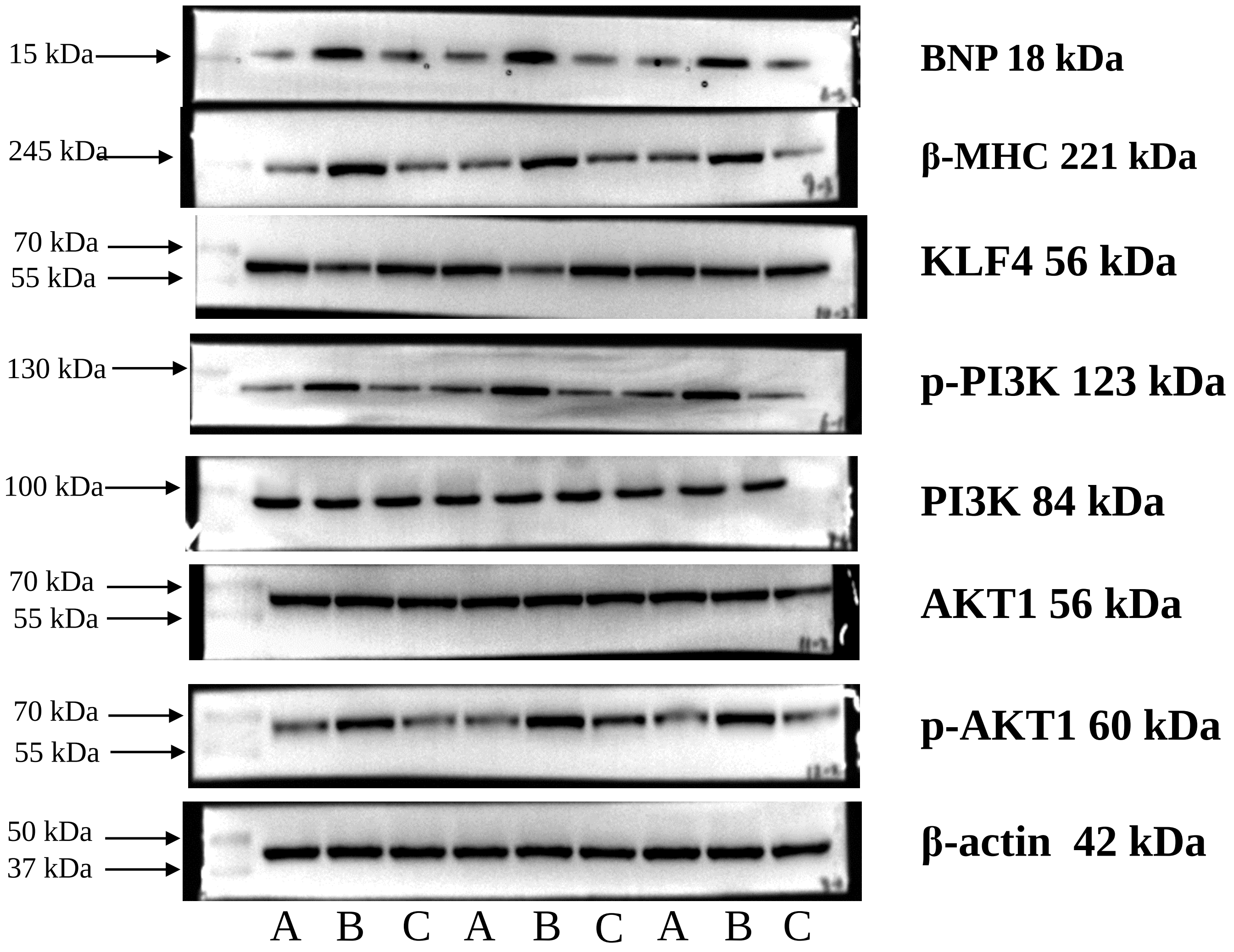

A, control
B, ISO
C, ISO+OT

Figure 8. Original western blot for three repeats

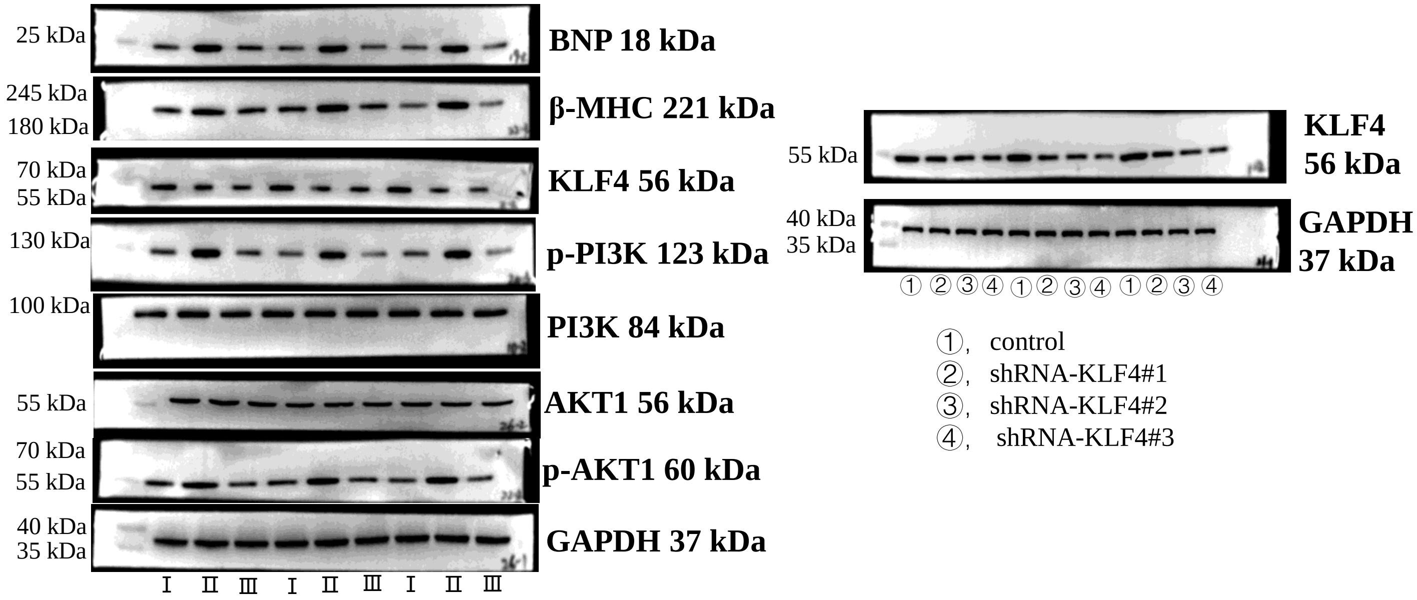

①, control
②, shRNA-KLF4#1
③, shRNA-KLF4#2
④, shRNA-KLF4#3

Ⅰ, ISO+OT+shRNA-NC
Ⅱ, ISO+OT+shRNA-KLF4
Ⅲ, ISO+OT+shRNA-KLF4+LY194002

Figure 6. Example of original western blot for three repeats

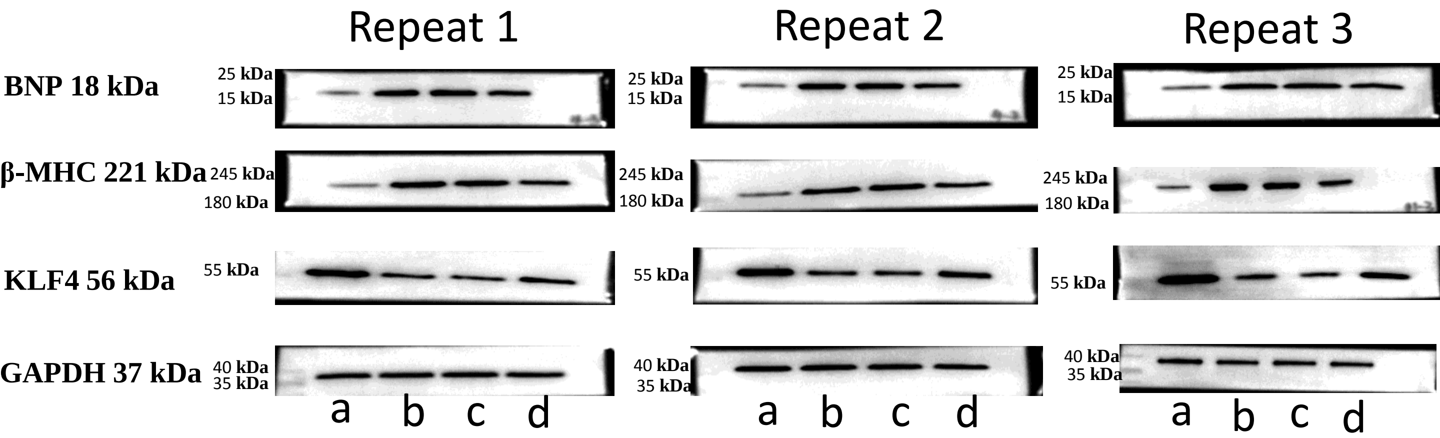

a, ISO+OT+shRNA-scramble
b, ISO+OT+shRNA-GAS5
c, ISO+OT+shRNA-GAS5+miR-375-3p inhibitor NC
d, ISO+OT+shRNA-GAS5+miR-375-3p inhibitor

Figure 7. Original western blot for three repeats

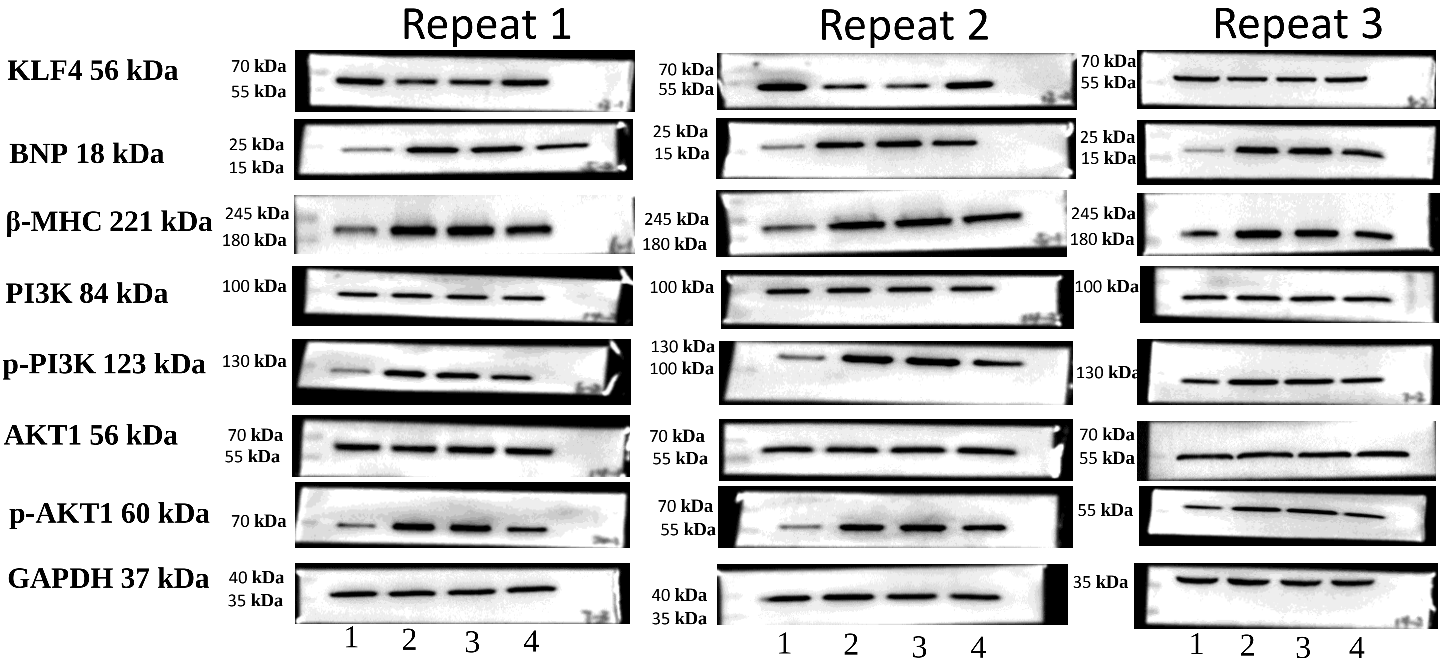

1, ISO+OT+miR-375-3p mimics NC
2, ISO+OT+miR-375-3p mimics
3, ISO+OT+miR-375-3p mimics+pcDNA-NC
4, ISO+OT+miR-375-3p mimics+pcDNA-KLF4
